# Antibacterial and wound-healing action of Ulmo honey (*Eucryphia cordifolia*) of differing degrees of purity

**DOI:** 10.3389/fvets.2023.1172025

**Published:** 2023-05-12

**Authors:** Mariela Muñoz, Mariano del Sol, Bélgica Vásquez

**Affiliations:** ^1^Centre of Excellence in Morphological and Surgical Studies, Universidad de La Frontera, Temuco, Chile; ^2^PhD Program in Morphological Sciences, Universidad de La Frontera, Temuco, Chile; ^3^Department of Basic Sciences, Faculty of Medicine, Universidad de La Frontera, Temuco, Chile

**Keywords:** natural product, Ulmo honey, melissopalynological analysis, wound healing, antibacterial action, re-epithelization

## Abstract

**Introduction:**

Antibacterial properties of honey vary according to its floral origin; few studies report the percentage of pollen types in honey, making it difficult to reproduce and compare the results. This study compares the antibacterial and wound-healing properties of three kinds of monofloral Ulmo honey with different percentages of pollen from *Eucryphia cordifolia*.

**Methods:**

The pollen percentage of the honey was determined by melissopalynological analysis, and they were classified into three groups: M1 (52.77% of pollen from *Eucryphia cordifolia*), M2 (68.41%), and M3 (82.80%). They were subjected to chemical analysis and agar diffusion test against *Staphylococcus aureus*. A total of 20 healthy adult guinea pigs (*Cavia porcellus*) of both sexes were randomly assigned to four groups for experimental burn skin wound (uninfected) production and treatment with Ulmo honey. On day 10 post-injury, biopsies were obtained, and histological analysis was performed to assess wound-healing capacity following the treatment with honey.

**Results:**

The chemical analysis showed that M3 differed significantly from M1 in terms of pH (*P* = 0.020), moisture (*P* = 0.020), total sugars (*P* = 0.034), and total solids (*P* = 0.020). Both strains of *Staphylococcus aureus* were susceptible to M1 and M2 at 40% w/v but were resistant to M3 at all concentrations. All groups (I–IV) were in the initial proliferative phase, with complete or partial re-epithelialization of the epidermis.

**Discussion:**

The antibacterial activity showed a wide range of variation in the different types of honey studied, with no significant differences between wound healing and pollen percentage in the groups studied. Higher pH and the absence of Tineo in M3 conferred a lower antibacterial capacity but not a lower wound healing capacity. Despite its variability in the percentage of *Eucryphia cordifolia* like primary pollen in Ulmo's monofloral honey, this has the same properties in relation to wound healing.

## 1. Introduction

Honey has been used for several centuries to treat wounds, and its use in humans (1, 2) and animals (3, 4) is currently being studied as an alternative treatment for wounds. Honey is a source of active compounds that are effective in a variety of bacterial infections caused by drug-resistant bacteria (5–9), which are a huge public health concern. It has healing properties, particularly in treating burns and wounds, and this has been demonstrated in animal experimentation (10–12) and in clinical trials (1, 2). The use of honey in the treatment of wounds in animals is a growing field due to its broad spectrum of activity against drug-resistant bacteria and its properties in the epithelialization process (3, 4). It has been shown that its high reducing sugar content, low water activity, high viscosity, high osmotic pressure, low pH, low protein content, and presence of hydrogen peroxide contribute to the antibacterial activity of honey (13, 14).

It is known that the botanical and geographic origin of honey, along with environmental factors inside the hive and during harvest and storage, affect its composition and antibacterial and wound-healing properties (15).

Experimental studies and clinical trials have demonstrated that honey promotes autolytic debridement, stimulates wound tissue growth and anti-inflammatory activity, and accelerates healing processes. Compared with topical agents, such as silver hydrofiber or silver sulfadiazine, honey is most effective at reducing the wound area and promoting re-epithelialization (16, 17). In addition, honey improves the outcome of wound healing by reducing the incidence and excessive formation of scars. The mechanisms of action of honey in wound healing are mainly due to its hydrogen peroxide content, high osmolality, acidity, non-peroxide factors, nitric oxide, and phenols (16, 17).

Despite the available evidence on the physicochemical characteristics of honey and its antibacterial and healing capacity, most studies on experimental and clinical models only report whether honey is monofloral or polyfloral and where it was obtained geographically, without giving more precise information on pollen characteristics (10, 18–21). It is generally considered that honey comes predominantly from a given botanical origin (monofloral) if the relative frequency of the primary pollen type of that taxon exceeds 45% (22). Chilean standard NCh2981.Of2005, dictated by the National Standards Institute in 2005, as well as the Mexican standard NOM-004-SAG/GAN-2018 on honey production and specifications, establishes that the botanical origin of honey must be determined by melissopalynological analysis, and for honey to be categorized as monofloral, it must have at least 45% pollen from a certain species (23–25); however, not all countries share these percentages. Although, in most cases, there is an agreement regarding the minimum percentage of pollen content required for the characterization of monofloral honey, it is not always the same value in different countries (26).

Due to its complex composition, even honey of the same botanical origin can exhibit different biological activities (27, 28). Therefore, if it is only reported that the honey is monofloral, without indicating its exact pollen percentage and the pollen range of the other floral sources (secondary pollen 16–45%, minor pollen 3–15%, and trace pollen 1–3%), the diversity of the types of honey used in the investigations brings with it a variability in the results and difficulties in the reproduction of the studies.

In Chile, there are several kinds of monofloral honey under study for their antibacterial and healing properties. Ulmo monofloral honey is obtained from the Ulmo tree (*Eucryphia cordifolia)*, a species native to southern Chile that flowers from the end of January until March.

Our hypothesis assumes that the higher the percentage of primary pollen, the greater the antibacterial activity and healing capacity of Ulmo honey. Therefore, the objective of this research was to conduct an exploratory study to determine and compare the antibacterial and wound-healing capacities, using three samples of monofloral Ulmo honey with different percentages of *Eucryphia cordifolia* pollen.

## 2. Materials and methods

### 2.1. Ethics

The protocol was approved by the Scientific Ethics Committee of the Universidad de La Frontera, file n°058-18. The determination of sample size was based on the bioethical principles of Russell and Burch's 3Rs for animal experimentation: replacement, reduction, and refinement. Our study was based on Cruz-Orive and Weibel's premise that five cases per group represent a sample that allows a statistically significant result. In general, in histology, quantitative methods are based on discrete random values, which often fall into a binomial distribution that is the basis of the binomial test of statistical significance; thus, the probability is calculated as *P* = (1/2)^*n*^, where n is the number of cases in the sample. Thus, with *n* = 5, a *P* = 0.03 is obtained, below the statistical level normally used as statistically significant (29, 30). The daily evaluation of animals was performed with the Daily Animal Supervision Protocol, Guide to Bioethical Aspects of Animal Experimentation, CONICYT (31), and the National Institutes of Health guide for the care and use of Laboratory animals (32).

### 2.2. Materials

Ulmo honey (*Eucryphia cordifolia)* was obtained from two different geographic origins in southern Chile: the Region of La Araucanía and the Region of Los Lagos. They were stored in the dark in plastic containers at room temperature (21–22°C). The honey used for the wound-healing studies was sterilized by gamma irradiation at 25 κGγ at the Chilean Nuclear Energy Commission (CCHEN).

### 2.3. Methods

#### 2.3.1. Melissopalynological analysis

Determination of the pollen percentage of *Eucryphia cordifolia* of Ulmo honey was performed by optical microscopy at the Universidad Austral of Chile according to NCh2981 (23, 24). In brief, 10 g of honey was diluted in 10 ml of distilled water and centrifuged at 2,500 rpm for 5 min, and the sediment was re-suspended in 0.1 ml of distilled water. Optical microscopy was used to observe the pollen grains which were identified using a palinotheque. The honey types were classified into three groups: monofloral honey M1, M2, and M3 with a purity percentage between 45 and 60%, 61 and 80%, and ≥81%, respectively.

#### 2.3.2. Chemical properties of the honey

A digital pH meter was used to determine the pH. To determine the moisture and ash content, 5 g of honey was weighed (m_0_) and liquefied in a melting pot, left in a stove at 105°C for 2 days to eliminate the water, and then weighed again (m_1_). The percentage of moisture was calculated by the equation as follows: (m_1_) – (m_0_) × 100. Then, honey without water was introduced in a melting pot (m_1_) to a muffle furnace at 600°C for 1 h and was allowed to cool in a desiccator and weighed again. The ash content was determined using the formula as follows: $Ash\;content\;\left(\frac{g}{100g}\right)=\frac{m_{1}\;-\;m_{2}}{m_{0}}x\;100$, where m_0_ = weight of the honey, m_1_ = weight of the pot + ash, and m_2_ = weight of the empty pot. The reducing sugars were determined through the reaction with 3.5-dinitrosalicylic acid (DNS), and for the determination of total sugars, a modified Miller's method (33) was used for the reducing sugars. The percentage of solids was determined by measuring the refraction index at 25°C in a refractometer (Leica^®^ Mark II INN, 1968).

#### 2.3.3. Agar diffusion test

A serial dilution was prepared for each Ulmo honey, from 40% w/v at 0.1% w/v. For the agar diffusion test, the CLSI methodology was used (34). Mueller Hinton Agar plates were inoculated with bacterial suspension, wells of 8.2 mm diameter were cut using a sterile punch (cork-borer set, Sigma–Aldrich Z165220), and 180 μl of each dilution of each honey was placed in each well. The plates were incubated at 35°C for 24 h, and the inhibition halos were measured with a Vernier caliper, including the diameter of the well. Interpretive criteria and a breakpoint for the honey were as follows: susceptible ≥20 mm, intermediate 15–19 mm, and resistant ≤ 14 mm; susceptible breakpoint 20 mm and resistant breakpoint 14 mm (35). A diffusion control of methylene blue was used. The strains tested were *Staphylococcus aureus* ATCC 25923 (methicillin-sensitive) and ATCC 43300 (methicillin-resistant).

#### 2.3.4. Animal model

Guinea pigs (*Cavia porcellus*) were used as animal models because they do not produce their own ascorbic acid and instead must obtain this vitamin from their diet. Ascorbic acid is essential to wound healing (36) because it is used in the production of collagen, which is a major component in wound healing and skin regeneration after injury. The guinea pig is also an excellent model for healing studies since its skin thickness remains constant once its body weight exceeds 450 g (37). A total of 20 healthy adult guinea pigs of both sexes were used, with an average weight of 833 g, maintained with pellets supplemented with ascorbic acid and vegetables *ad libitum* under controlled temperature conditions, environmental noise, and light-dark cycles (12–12 h) in the Center of Excellence in Morphological and Surgical Studies (CEMyQ) at the Universidad de La Frontera. The guinea pigs were randomized to four experimental groups by simple randomization. Group distribution was blinded until the end of the study. The participants were divided into the following groups: Group I—animals treated with M1 honey, Group II—animals treated with M2 honey, Group III—animals treated with M3 honey, and Group IV (control)—animals treated with hydrogel-tull (Urgo Hydrogel, Urgo Medical).

#### 2.3.5. Wound model

The wound in each animal was created according to the protocol previously described (38). An injury was made on the back by contact with a hot circular metal object with a diameter of 1 cm for 3 s, this was done under deep sedation effects (ketamine 40 mg/kg, xylazine 5 mg/kg, and 0.05 atropine mg/kg) intraperitoneally, and the animal's recovery was done by thermoregulation. The treatments consisted of cleaning the wound with warm physiological serum using a syringe 10 cm from the injury of the conscious animal. Then, a gauze was impregnated with M1, M2, and M3 Ulmo honey, or hydrogel-tull, according to the corresponding group. An escharotomy was performed on each wound to facilitate the impregnation of the treatments until the necrotic tissue was loosened. All the animals were treated and evaluated daily until 10 days post-injury (38, 39). The animal monitoring protocol included scoring for appearance, feed and water intake/weight loss, clinical signs, handling, and spontaneous behavior. Each parameter had a maximum score of 4 points, with the protocol having a maximum total score of 20. Up to 4 total points were considered normal, and from 5 points, the parameter was carefully monitored, and analgesia was evaluated. All study animals averaged < 4 total points. Therefore, according to the protocol of daily monitoring of animals, no analgesia was necessary during the study period.

#### 2.3.6. Biopsy and histological processing

On post-injury day 10, a biopsy was obtained from the burn area. For this, the skin was washed with an antiseptic; then, 2% lidocaine was injected subcutaneously, and the biopsy was extracted with a 10 mm punch until it reached the deep dermis and then proceeded to suture the resulting injury. The samples were fixed in a 10% buffered formalin for 48 h, and the samples were prepared for sectioning and histological staining with hematoxylin–eosin (HE). The slides were visualized in an optical microscope (Leica^®^ DM 2000 LED) and photographed with a digital camera (Leica^®^ MC 170 HD).

The quantitative histological analysis was performed according to the burn wound healing scale described by Mehrabani et al. (40) and Hazrati et al. (18). Table 1 shows the parameters evaluated.

**Table 1:** The scoring system of the histological changes in burn-wound healing.

**Score**	**Re-epithelization**	**Granulation**	**Inflammatory cells**	**Angiogenesis**
0	Absence of epithelial proliferation in ≥ 70% of the tissue.	Immature and inflammatory tissue in ≥ 70% of the tissue.	13–15 inflammatory cells per histological field.	Absence of angiogenesis, presence of congestion, hemorrhage, and edema.
1	Poor epidermal organization in ≥ 60% of the tissue.	Sparse immature and inflammatory tissue in ≥ 60% of the tissue.	10–13 inflammatory cells per histological field.	1–2 vessels per site, edema, hemorrhage, and congestion.
2	Incomplete epidermal organization in ≥ 40% of the tissue.	Moderate remodeling in ≥ 40% of the tissue.	7–10 inflammatory cells per histological field.	3–4 vessels per site, moderate edema, and congestion.
3	Moderate epithelial proliferation in ≥ 60% of the tissue.	Layer of coarse granulation and well-formed collagen matrix in ≥ 60% of the tissue.	4–7 inflammatory cells per histological field.	5–6 vessels per site, slight edema, and congestion.
4	Complete epidermal remodeling in ≥ 80% of the tissue.	Complete tissue organization in ≥ 80% of the tissue.	1–4 inflammatory cells per histological field.	More than seven vessels per site arranged vertically toward the epithelial surface.

#### 2.3.7. Statistical methods

For the data analysis, IBM SPSS version 26 statistical software was used. In all the analyses, *P* < 0.05 was considered statistically significant. Variables are presented as mean ± standard deviation for the chemical parameters, inhibition halo, the median, lower and upper limit range for the histological scale score. For chemical properties analyses, agar diffusion test, and histological scoring system, normality tests of each group using Shapiro–Wilk test were conducted individually. To identify whether there were significant differences between the groups in the chemical properties of the different types of honey, the Kruskal–Wallis test was used with Dunn's test of multiple comparisons. To identify whether there were significant differences between the groups in the agar diffusion test, the two-way ANOVA and Tukey's test of multiple comparisons were used. To evaluate whether there were significant differences between the groups in the total score of the histological healing scale, a one-way ANOVA was used with a Tamhane *post-hoc* test. The Kruskal–Wallis test was performed to evaluate significant differences between the groups for each parameter of the healing scale.

## 3. Results

### 3.1. Melissopalynological analysis

According to the percentage of each pollen form with respect to the total pollen number (41), M1 presented a percentage of 52.77%; M2, 68.41%; and M3, 82.80% of *Eucryphia cordifolia*. The low-frequency pollen with the highest percentage in M1 and M2 was the Big trefoil (*Lotus uliginosus*), with 8.46 and 14.0%, respectively, and in M3, it was Tiaca (*Caldcluvia paniculata*) with 9.34%. The pollen from the Chilean myrtle (*Luma apiculata*) was present in all three types of honey; however, in M1 and M2, it was a low-frequency category of pollen, with 6.30 and 12.80%, respectively, and in M3, in the trace category with 1.68% (Supplementary Table 1).

### 3.2. Chemical properties of the honey

Table 2 shows the average and standard deviation of the six chemical parameters determined in M1, M2, and M3. The M3 presented significant differences with M1 in pH (*P* = 0.020), moisture (*P* = 0.022), total sugars (*P* = 0.034), and total solids (*P* = 0.020).

**Table 2:** Chemical parameters of the three samples of Ulmo honey (*Eucryphia cordifolia*).

**Parameters**	**M1**	**M2**	**M3**	***P*-value**
pH	3.73 ± 0.03	3.81 ± 0.01	4.55 ± 0.01^*^	0.020
Moisture (%)	21.34 ± 0.38	18.74 ± 0.17	16.31 ± 0.34^*^	0.022
Ashes (g/100 g)	0.18 ± 0.01	0.18 ± 0.09	0.07 ± 0.01	0.059
Reducing sugars (g/100 g)	70.36 ± 1.24	72.10 ± 4.06	65.38 ± 1.49	0.155
Total sugars (g/100 g)	75.00 ± 2.70	79.79 ± 2.37	83.30 ± 2.03^*^	0.034
Total solids (%)	76.20 ± 0.17	78.47 ± 0.06	80.03 ± 0.06^*^	0.020

M1: 52.77%, M2: 68.41%, and M3: 82.80% of *Eucryphia cordifolia*. The results show the mean of triplicates and standard deviation.

^*^Significant difference (P < 0.05) with group M1.

### 3.3. Agar diffusion test

The agar diffusion test showed that the greater the concentration of the honey, the greater the antibacterial action on both strains. According to the CLSI standards, the dilution at 40% w/v of M1 and M2 shows that both strains were sensitive to the effects of M1 and M2. By contrast, M3 presented a smaller inhibition halo in both strains of *Staphylococcus aureus*, which, according to CLSI standards (31), shows that these strains were resistant to the antibacterial action of M3. Values are shown in Supplementary Tables 2, 3.

**Figure 1:**
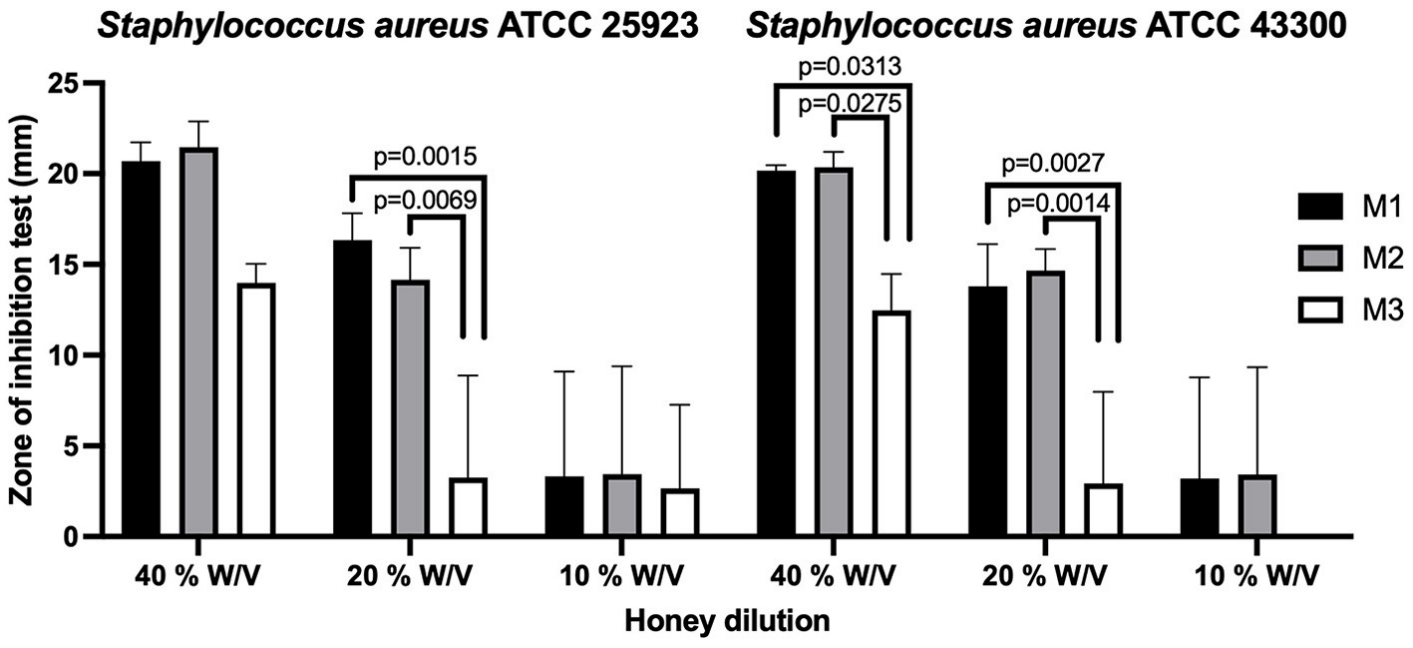
The agar diffusion test in Ulmo honey types M1, M2, and M3 for *Staphylococcus aureus* ATCC 25923 (methicillin-sensitive) and *Staphylococcus aureus* ATCC 43300 (methicillin-resistant). Significant statistical differences *P* < 0.05.

According to CLSI standards (35), in the dilution at 20% w/v, M1 had intermediate antibacterial action for *Staphylococcus aureus* ATCC 25923 and was resistant to the *Staphylococcus aureus* ATCC 43300 methicillin-resistant strain. The 20% w/v dilution of M2 had a very low antibacterial effect against *Staphylococcus aureus*, and both strains were resistant to this dilution. At the 10% w/v dilution, all types of honey showed little or no antibacterial action on both *Staphylococcus aureus* isolates, and both strains were resistant. The statistical differences between the groups are presented in Figure 1.

### 3.4. Histological analysis

#### 3.4.1. Normal guinea pig skin

**Figure 2:**
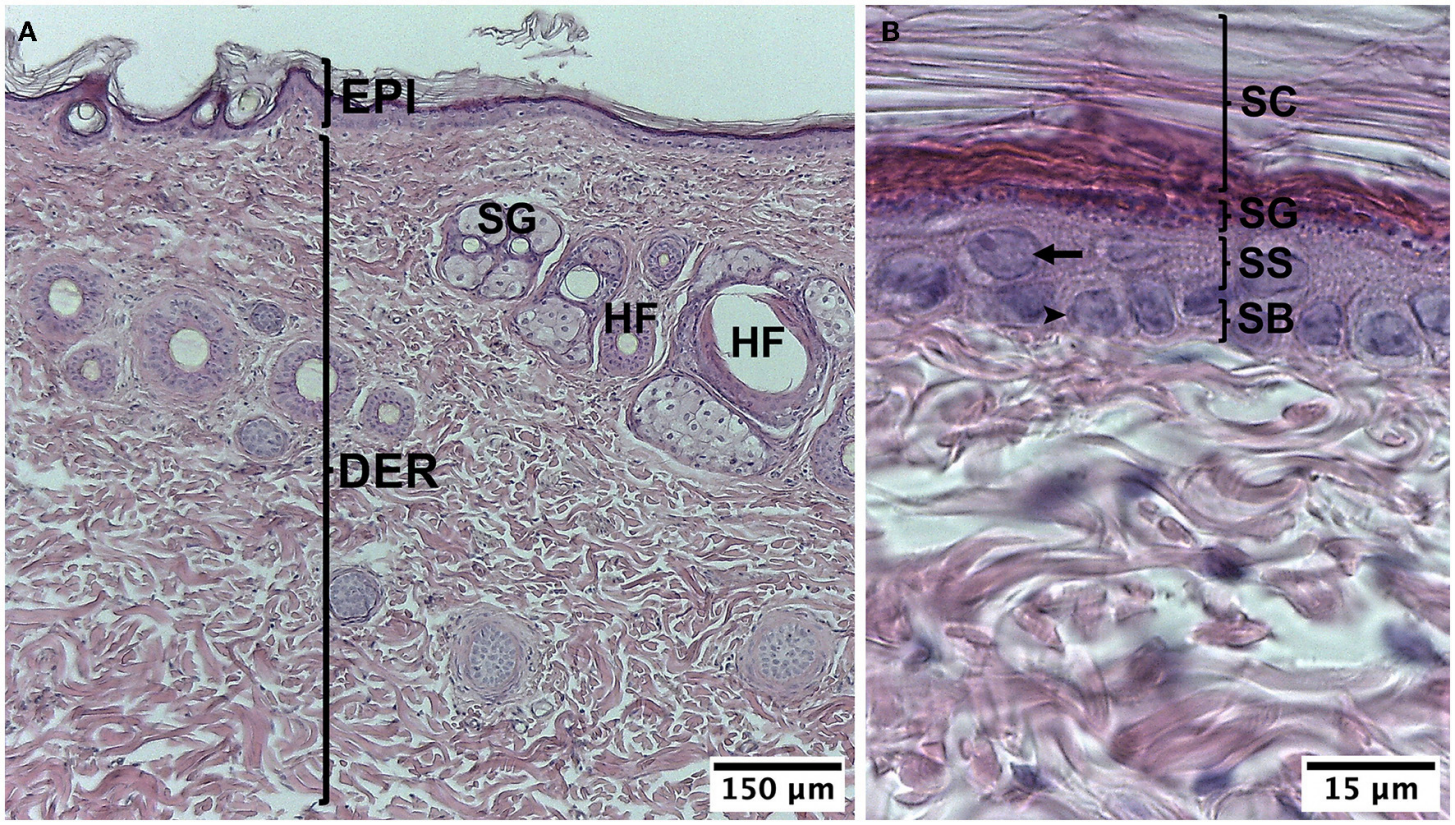
Normal histology of guinea pig (*Cavia porcellus*). **(A**) Epidermis and dermis. **(B)** Strata of the epidermis. EPI, epidermis; DER, dermis; SG, sebaceous gland; HF, hair follicle; SC, stratum corneum; SG, stratum granulosum; SS, stratum spinosum; SB, stratum basale; arrow, keratinocyte, arrow point; cell of the stratum basale. HE staining.

The skin on the back of the guinea pig was characterized as having a thin epidermis, which was made up of the stratum corneum, with some keratinized layers, the stratum granulosum with elongated cells arranged parallel to the surface, whose cytoplasm has abundant basophil granules, the stratum spinosum with one or two layers of keratinocytes, and the stratum basale with cells whose round or oval nuclei were neatly arranged on the basal lamina. In the dermis, the stratum papillare was unclear because the dermoepidermal junction did not have sinuous undulations; the stratum reticulare, which was thicker, was made up of irregular dense connective tissue, which had hair follicles and sebaceous glands. More deeply, the subcutaneous tissue or hypodermis had abundant adipose cells (Figure 2).

#### 3.4.2. Group I

**Figure 3:**
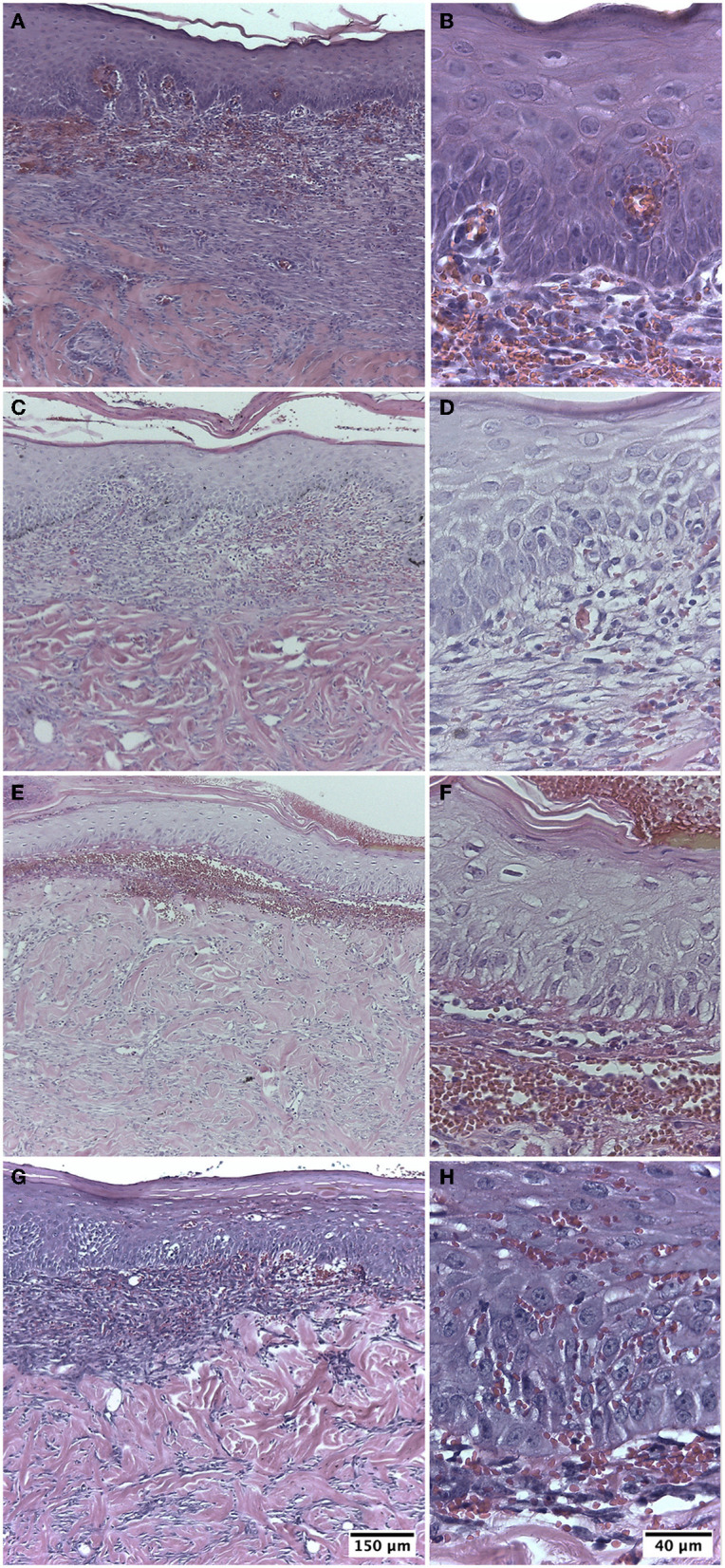
Biopsy of guinea pig skin (*Cavia porcellus*) on post-injury day 10. **(A, B)** Treatment with Ulmo honey M1. **(C, D)** Treatment with Ulmo honey M2. **(E, F)** Treatment with Ulmo honey M3. **(G, H)** Treatment with hydrogel-tull. HE staining.

The biopsies from group I were in the initial proliferative phase with a fibroblastic reaction on post-injury day 10. In 40% of the biopsies, complete epidermoid remodeling was visualized, with increased thickness and the presence of keratin. In the superficial part of the dermis, vascular neoformation, inflammatory cells, and edema were observed in some of the samples. The connective tissue presented abundant fibroblasts and regularly arranged collagen fibers. Eschar was noted in two biopsies (Figures 3A, B).

#### 3.4.3. Group II

This group presented characteristics similar to group I, with an initial proliferative phase with a fibroblastic reaction at post-injury day 10. In total, 60% of the biopsies showed complete re-epithelialization with the presence of keratin. In the superficial part of the dermis, neoformation of blood vessels, inflammatory cells, and some of the sample's edema were observed (Figures 3C, D).

#### 3.4.4. Group III

The biopsies were in the initial proliferative phase on post-injury day 10. In total, 40% of them showed moderate epithelial proliferation in ≥60% of the tissues. In the superficial part of the dermis, edema, inflammatory cells, and some blood vessels in formation were observed. Two samples presented eschar (Figures 3E, F).

#### 3.4.5. Group IV

The biopsies presented an initial proliferative phase with a fibroblastic reaction on post-injury day 10. In total, 20% of the biopsies showed complete epithelial regeneration with the presence of keratin. The scar dermis presented loose connective tissue with collagen fibers, neoformation of blood vessels, and edema. In three samples, it was still possible to observe the presence of eschar (Figures 3G, H).

#### 3.4.6. Burn wound healing scale

Table 3 shows the percentage of animals that were categorized to each score of the four histological parameters. The median and the lower and upper range of burn wound healing score for M1 was 5 (2–11), M2 was 10 (1–12), M3 was 6 (3–9), and M4 was 5 (1–10). Statistical analysis showed no significant differences between the four groups (*P* = 0.619). No significant differences were observed in the medians for re-epithelialization (*P* = 0.644), granulation (*P* = 0.190), inflammatory (*P* = 0.475), and angiogenesis (*P* = 0.343).

**Table 3:** Percentage of the histological parameters on the burn wound healing scale.

**Parameters**	**Score**	**Group frequency (%)**			
		**Group I**	**Group II**	**Group III**	**Group IV**
Re-epithelization	0	0	20	0	40
	1	60	20	20	0
	2	0	0	40	40
	3	0	0	40	0
	4	40	60	0	20
Granulation	0	20	20	0	40
	1	40	20	40	0
	2	0	0	60	40
	3	40	60	0	20
	4	0	0	0	0
Inflammatory cells	0	20	20	40	80
	1	60	40	40	20
	2	20	40	20	0
	3	0	0	0	0
	4	0	0	0	0
Angiogenesis	0	0	0	0	0
	1	40	40	80	80
	2	40	40	20	20
	3	20	20	0	0
	4	0	0	0	0

Group I: treated with M1 (52.77%), group II: treated with M2 (68.41%), group III: treated with M3 (82.80%), and group IV: treated with hydrogel-tull, positive control. The histological scoring system ranges between 0 and 4, where 4 is the best score for each histological parameter.

## 4. Discussion

This research was an exploratory study aimed at determining whether the antibacterial and wound-healing properties of Ulmo honey are related to the percentage of pollen from *Eucryphia cordifolia*.

The pollen analysis showed a greater diversity of secondary species in the M1 honey, and these pollen characteristics may be interesting regarding biological properties. For example, the presence of Tineo (*Weinmannia trichosperma*) has been associated with a high content of total phenols (42), which could explain, in part, the greater antibacterial activity of M1 honey compared to M2 and M3 types of honey that do not have Tineo pollen, unlike the M1 type, which has 3.31% of this pollen. The same study (42) showed that the Ulmo honey with a high degree of purity and the presence of low-frequency pollen or trace pollen of Chilean myrtle (*Luma apiculata) or* big trefoil (*Lotus pedunculatus*) present high activity against *Staphylococcus aureus*. In our study, the three types of honey presented Chilean myrtle *(Luma apiculata);* however, the M3 honey presented a lower percentage of this pollen with only 1.68%, which coincides with lower antibacterial activity. Melissopalynological analysis shows that Big trefoil (*Lotus pedunculatus*), cat's ear (*Hypochaeris radicata*), eucalyptus (*Eucalyptus* spp.), field mustard (*Brassica campestris*), and white clover (*Trifolium repens*) are only present in M1 and M2 types of honey, this may be interesting if we observe that the antibacterial activity of these two types of honey was more similar compared to M3 honey.

The results of the chemical analysis demonstrated that M3 differed significantly from M1 in terms of pH (*P* = 0.020), a recognized parameter that affects the antibacterial capacity of the honey, and its variation depends on the honey's botanical origin (43); thus, the pH values of floral honey are usually between 3.4 and 5.5. The pH value is related to the existence and growth of microorganisms. EU Council Directive 2001/110/EC (44) does not impose a maximum value allowed for the pH of honey; however, a low pH avoids microbiological deterioration, this is in accordance with our results, while honey with a higher pH (M3) has less antibacterial activity, and honey with the lowest pH has the highest antibacterial activity (M1). It was reported (45) that samples of Ulmo honey had a pH of 4.05 for honey with a low percentage of Ulmo (51.93%) and a pH of 3.97 for honey with a high percentage of Ulmo (84.25%). This difference compared to our study could be due to the pH varying between different types of honey of the same floral origin but in a different geographic area. Other authors (46) reported that the monofloral acacia honey from central Serbia had a pH of 4.52 ± 0.02, while another group (47) reported that monofloral acacia honey from Romania had a pH of 3.80 ± 0.02 and from Poland had a pH of 3.58 ± 0.05. In both studies, the exact percentage of acacia pollen was not indicated, only that they were monofloral honey. There is also a significant difference between the different types of honey in moisture (*P* = 0.022), total sugars (*P* = 0.034), and total solids (*P* = 0.020), demonstrating that each honey has its own composition according to its floral source, which is related to its different properties (47).

Studies have shown that in Chilean honey, phenolic compounds play an important role in its antibacterial properties (42, 48). For Ulmo honey, Acevedo et al. (49) demonstrated that a broad range of volatile and non-volatile/semi-volatile components rich in benzene derivatives could partially explain its antibacterial properties. Velásquez et al. (42) compared the phenolic composition and antioxidant and antimicrobial activities of eight samples of Ulmo honey. The samples used had a predominance of *Eucryphia cordifolia* between 86.6 and 96.8%, which, in our study, would correspond to M3, with a lower percentage of *Eucryphia cordifolia* pollen not evaluated, so their conclusions do not necessarily apply to all the types of Ulmo monofloral honey.

The presence of low-frequency pollens could also influence the antibacterial activity of Ulmo honey. Velásquez et al. (42) found no correlation between the antibacterial activity against *Staphylococcus aureus* and the botanical origin of the primary pollen because the predominance of *Eucryphia cordifolia* was not significantly different among the samples (range 92.2 ± 3.9%). Nevertheless, they observed that the inhibition of *Staphylococcus aureus* was directly related to the amount of Ulmo and Tineo (*Weinmannia trichosperma*) nectar and the inhibition of *Escherichia coli* strains with the amount of Ulmo and Chilean myrtle pollen.

Furthermore, they observed that the inhibition of *Pseudomonas aeruginosa* had a stronger relationship with the Tineo content than with the Ulmo content. In our study, the greatest inhibition halos of *Staphylococcus aureus* (methicillin-sensitive and methicillin-resistant), at 40 and 20% w/v, occurred in M1 and M2, respectively. These types of honey mainly contained Big trefoil (*Lotus pedunculatus*) and Chilean myrtle (*Luma apiculata*), whereas M3 presented Tiaca (*Caldcluvia paniculata*) and Pussy willow (*Salix caprea*). This suggests that the percentage of dominant pollen of monofloral honey might not, in itself, be an important factor in antimicrobial activity but that the contribution of secondary pollen, low-frequency, or trace pollen in monofloral honey could also be acting synergically for the antibacterial effect of the honey.

Sherlock et al. (8) used agar diffusion and minimum inhibitory tests to demonstrate the antibacterial effects of Manuka and Ulmo honey (90% *Eucryphia cordifolia*). Ulmo honey was the most effective against *Staphylococcus aureus* ATCC 43300, although slightly less effective against *Escherichia coli* and *Pseudomonas aeruginosa*. It was also established that the elimination of the hydrogen peroxide activity in Ulmo honey reduced its antimicrobial activity against *Staphylococcus aureus*.

In our study, the antibacterial activity of three Ulmo honey samples was tested by the agar diffusion test, and it was observed that the activity depends on the concentration. M1 and M2 showed antibacterial action against methicillin-sensitive (ATCC 25923) and methicillin-resistant (ATCC 43300) *Staphylococcus aureus* in the dilution of 40% w/v of honey; M3 was resistant at the same dilution. Our results are different from those observed by Sherlock et al. (8), who reported antibacterial activity against methicillin-resistant *Staphylococcus aureus* (ATCC 43300) at 50, 25, and 12.5% v/v in Ulmo honey with 90% *Eucryphia cordifolia*. These differences could be due to the influence of other factors that also affect antibacterial activity (47, 50–52).

In relation to the inhibition of *Staphylococcus aureus* at 40 and 20% w/v and the chemical characteristics of the different samples of honey, it was observed that the honey varieties with lower pH (M1 and M2) presented greater inhibitory action. These results were to be expected since studies have shown that the acidity of honey contributes to its antibacterial activity (53, 54); it is, nevertheless, important to consider that it is not sufficient to inhibit the growth of many bacterial species when it is diluted in food or bodily fluids (55). Similarly, the high sugar content in the honey in combination with a low moisture content can cause osmotic stress, which prevents the deterioration of the honey produced by microorganisms (46, 56). Our results show that, although M3 has a high sugar content and low moisture content, it had the lowest antibacterial activity, so the characteristics of honey should be evaluated as a whole and not separately.

In this study, we were able to demonstrate that the wound-healing capacity of the three samples of Ulmo honey used, with a percentage of *Eucryphia cordifolia* between 52.77 and 82.80%, revealed no significant differences in the histological parameters evaluated with the burn wound healing scale. Furthermore, the three samples of honey used did not show significant differences compared with the hydrogel-tull positive control. Hydrogel-tull is an autolytic debrider composed of thickening and moisturizing polymers, widely used in wound treatment; however, it could allow the growth of gram-negative bacteria, and its use is limited in high-exuding wounds (57). On the contrary, honey has a set of properties that contribute to wound healing, highlighting its antibacterial and anti-exudative actions (16, 17), which makes honey advantageous compared to hydrogel-tull. A previous study by our research group (38) showed that honey alone, without ascorbic acid supplementation, showed comparable histological parameters with hydrogel-tull treatment; however, in this study, only honey with an Ulmo percentage of 90% *Eucryphia cordifolia* was evaluated. In the present study, our research question was whether honey with a higher percentage of *Eucryphia cordifolia* pollen had a better action in wound healing compared to honey that, being monofloral, had a lower percentage of *Eucryphia cordifolia* pollen.

The wound-healing capacity of different types of honey used in our study was similar to that reported by Schencke et al. (39), who used Ulmo honey with 90% *Eucryphia cordifolia*, within an initial proliferative phase on post-injury day 10, without epidermal regeneration. In the scar dermis, they observed neoformation of blood vessels, abundant cellularity, and active fibroblasts. Another study by these same researchers (38) used honey with 88.03% Ulmo (*Eucryphia cordifolia*), whose main secondary pollen species were Big trefoil (*Lotus uliginosus*) and Chilean myrtle (*Luma apiculata*), with 6.18 and 2.68%, respectively. This suggests that regardless of the honey pollen percentage used (≥45% *Eucryphia cordifolia*), the wound-healing capacity was similar among them. This, from the clinical point of view, is important because one of the main misgivings about the use of natural products is their variation between seasons. This seasonal variation can affect the presence and percentages of primary, secondary, minor, and trace pollen. For this reason, the information obtained from our research is important, since it shows that the variability of the percentage of primary pollen would not be a limitation in the results of wound healing, which represents an advance in the standardization of the use of honey as treatment in wound healing.

The use of honey as a topical treatment in animals and humans is recommended due to its stability, low health risk, easy application, and absence of risk of selecting bacterial resistance (38), and these advantages are the main arguments in favor of the application of honey for the treatment of wounds. Although advanced dressings, such as hydrogel-tull, are highly effective in wound healing and can obtain additional antibacterial properties by loading antibiotics specifically, they are expensive compared to honey-based dressings and also have the risk of the emergence of antibacterial resistance.

Finally, we believe that, in addition to molecular studies and more detailed information on the chemical composition of honey, future studies should include a collection of honey from different geographic locations and expand the number of samples to better understand how its pollen composition affects the honey's antibacterial action and wound-healing capacity.

## 5. Conclusion

In this study, three samples of monofloral honey from Ulmo with different percentages of *Eucryphia cordifolia* were compared for the first time in terms of their chemical characteristics, antibacterial activity, and wound healing of skin injured by burns.

The results of the chemical analyses showed variability among the three types of honey analyzed, with a greater difference between honey varieties M1 and M3. We can infer that the higher pH and the absence of Tineo in M3 confer a lower antibacterial capacity but not a lower wound-healing capacity, although, for more robustness of the results, a greater number of samples should be analyzed.

The importance of this exploratory study is to provide the first-time evidence that Ulmo's monofloral honey has the same properties in relation to wound healing despite its variability in the percentage of *Eucryphia cordifolia* like primary pollen. Therefore, the use of products based on monofloral honey is recommended as a treatment for wounds because it is a natural, stable, effective, and low-cost product; however, more research is required in the field.

## Data availability statement

The raw data supporting the conclusions of this article will be made available by the authors, without undue reservation.

## Ethics statement

The animal study was reviewed and approved by Scientific Ethics Committee of the Universidad de La Frontera, with approval number: 058-18.

## Author contributions

MM: conceptualization, formal analysis, investigation, methodology, writing—original draft, and visualization. MS: conceptualization, funding acquisition, project administration, supervision, and writing—reviewing and editing. BV: data curation, formal analysis, methodology, visualization, writing—original draft, and writing—reviewing and editing. All authors contributed to the article and approved the submitted version.
