# Supplementary material for: Antibacterial and wound-healing action of Ulmo honey (Eucryphia cordifolia) of differing degrees of purity
Source: Front Vet Sci. 2023 May 12;10:1172025. doi: 10.3389/fvets.2023.1172025 (PMC10213365; doi:10.3389/fvets.2023.1172025)
Supplement: Supplementary file 1 [file Table_1.DOCX]

Supplementary Material

Antibacterial and Wound healing Action of Ulmo Honey (*Eucryphia cordifolia*) of Differing Degrees of Purity

Mariela Muñoz^1,2^, Mariano del Sol^1,2,*^ and Bélgica Vásquez^3,*^

*** Correspondence:**

Mariano del Sol: mariano.delsol@ufrontera.cl

Bélgica Vásquez: belgica.vasquez@ufrontera.cl

**Table 1 (supple).** Melissopalynological analysis of the three samples of Ulmo honey (*Eucryphia cordifolia*).

| **Honey** | **Common Name** | **Scientific Name** | **% pollen grain** |
| --- | --- | --- | --- |
| M1 | **Ulmo** | ***Eucryphia cordifolia*** | **52.77** |
|  | Big trefoil | *Lotus uliginosus* | 8.46 |
|  | Chilean myrtle | *Luma apiculata* | 6.30 |
|  | Cat’s ear | *Hypochoeris radicata* | 4.69 |
|  | Blueweed | *Echium vulgare* | 4.41 |
|  | Bull thistle | *Cirsium vulgare* | 4.14 |
|  | Eucalyptus | *Eucaliptus spp.* | 3.59 |
|  | Tiaca | *Caldcluvia paniculata* | 3.31 |
|  | Tineo | *Weinmannia trichosperma* | 3.31 |
|  | Field mustard | *Brassica campestris* | 3.03 |
|  | White clover | *Trifolium repens* | 3.03 |
|  | Chilean hazel | *Gevuina avellana* | 2.86 |
| M2 | **Ulmo** | ***Eucryphia cordifolia*** | **68.41** |
|  | Big trefoil | *Lotus uliginosus* | 14.00 |
|  | Chilean myrtle | *Luma apiculata* | 12.80 |
|  | White clover | *Trifolium repens* | 2.94 |
|  | Field mustard | *Brassica campestris* | 0.63 |
|  | Ivy of Uruguay | *Cissus striata* | 0.55 |
|  | Cat’s ear | *Hypochoeris radicata* | 0.31 |
|  | Eucalyptus | *Eucaliptus spp.* | 0.31 |
| M3 | **Ulmo** | ***Eucryphia cordifolia*** | **82.80** |
|  | Tiaca | *Caldcluvia paniculata* | 9.34 |
|  | Pussy willow | *Salix caprea* | 4.48 |
|  | Chilean myrtle | *Luma apiculata* | 1.68 |
|  | Petra | *Myrceugenia exsucca* | 1.12 |
|  | Ribwort plantain | *Plantago lanceolata* | 0.56 |

**Table 2 (supple).** Agar diffusion test. Inhibition halos of bacterial growth for *Staphylococcus aureus* ATCC 25923.

| Dilution | **M1 Honey** | | **M2 Honey** | | **M3 Honey** | |
| --- | --- | --- | --- | --- | --- | --- |
|  | Mean (mm) | SD | Mean (mm) | SD | Mean (mm) | SD |
| 40 % | 20.69 | 1.02 | 21.45 | 1.42 | 13.97 | 1.05 |
| 20 % | 16.33 | 1.49 | 14.15 | 1.75 | 3.25 | 5.62 |
| 10 % | 3.33 | 5.76 | 3.44 | 5.95 | 0 | 0 |
| 5 % | 0 | 0 | 0 | 0 | 0 | 0 |

**Table 3 (supple).** Agar diffusion test. Inhibition halos of bacterial growth for Staphylococcus aureus ATCC 43300.

| Dilution | **M1 Honey** | | **M2 Honey** | | **M3 Honey** | |
| --- | --- | --- | --- | --- | --- | --- |
|  | Mean (mm) | SD | Mean (mm) | SD | Mean (mm) | SD |
| 40 % | 20.17 | 0.17 | 20.34 | 0.49 | 12.46 | 1.15 |
| 20 % | 13.78 | 1.35 | 14.65 | 0.68 | 2.92 | 2.92 |
| 10 % | 3.21 | 5.13 | 3.41 | 5.91 | 0 | 0 |
| 5 % | 0 | 0 | 0 | 0 | 0 | 0 |
